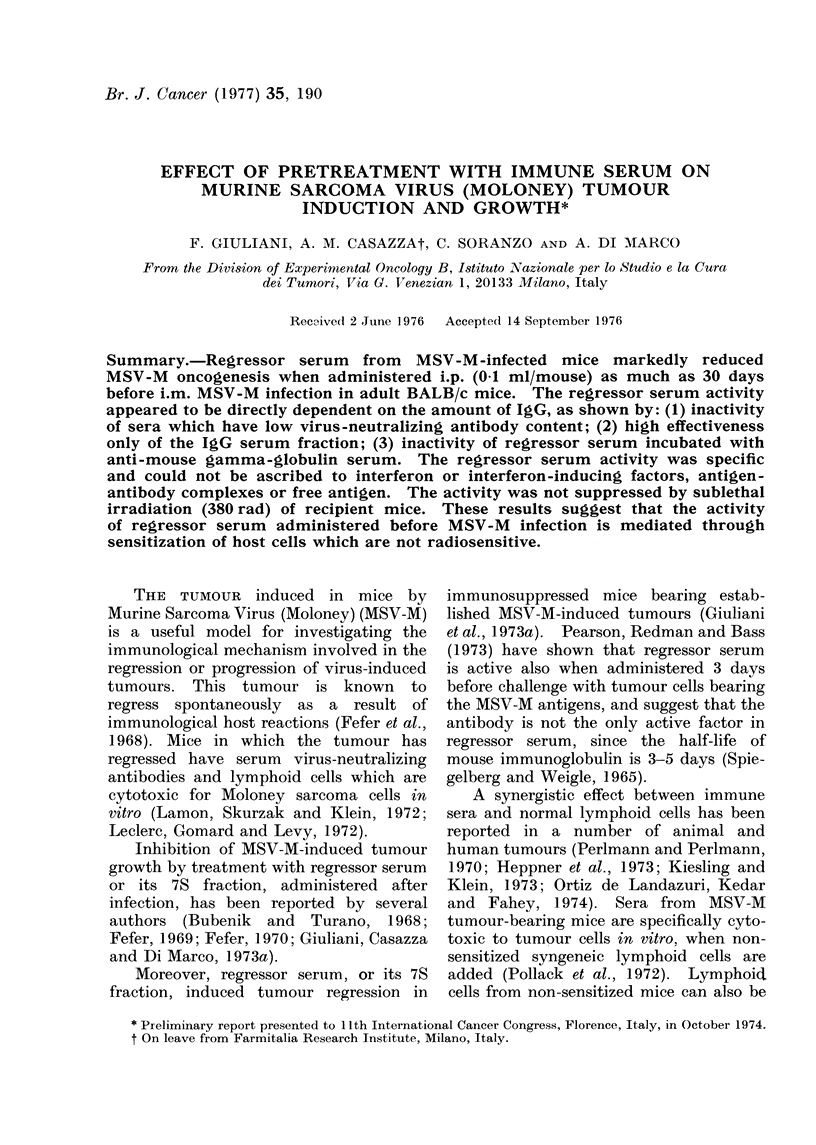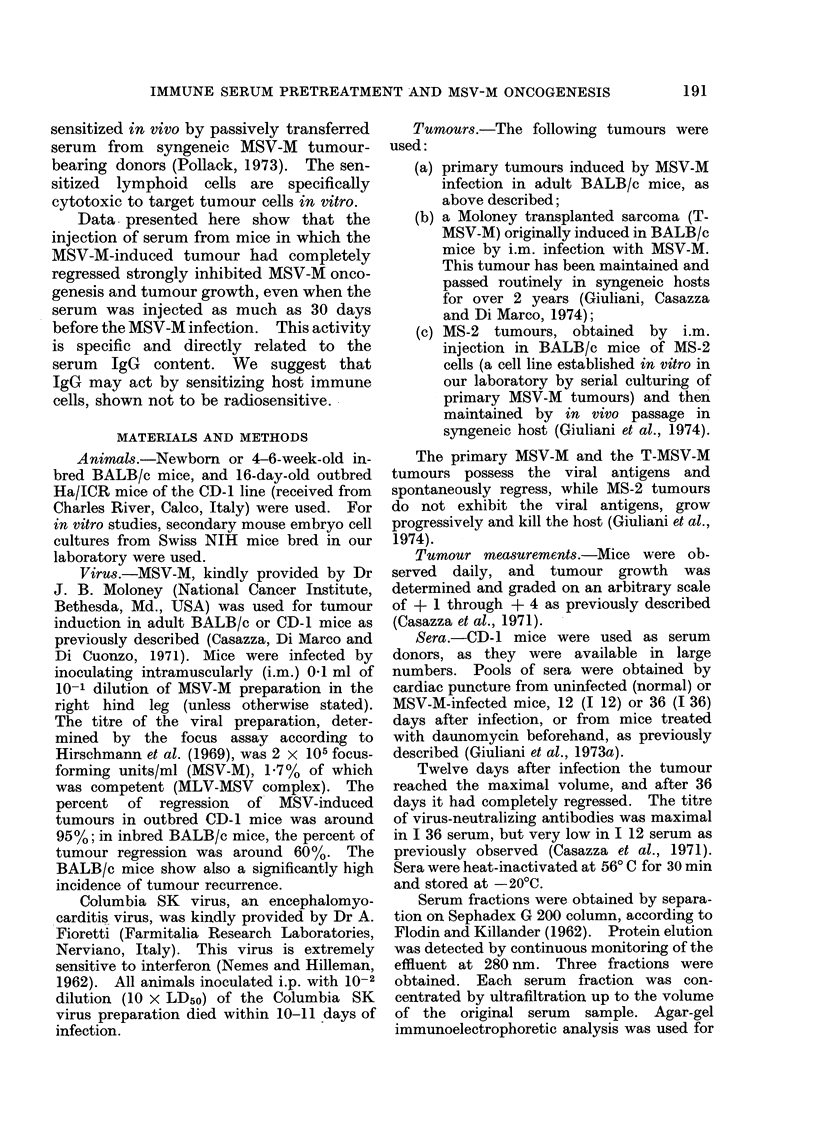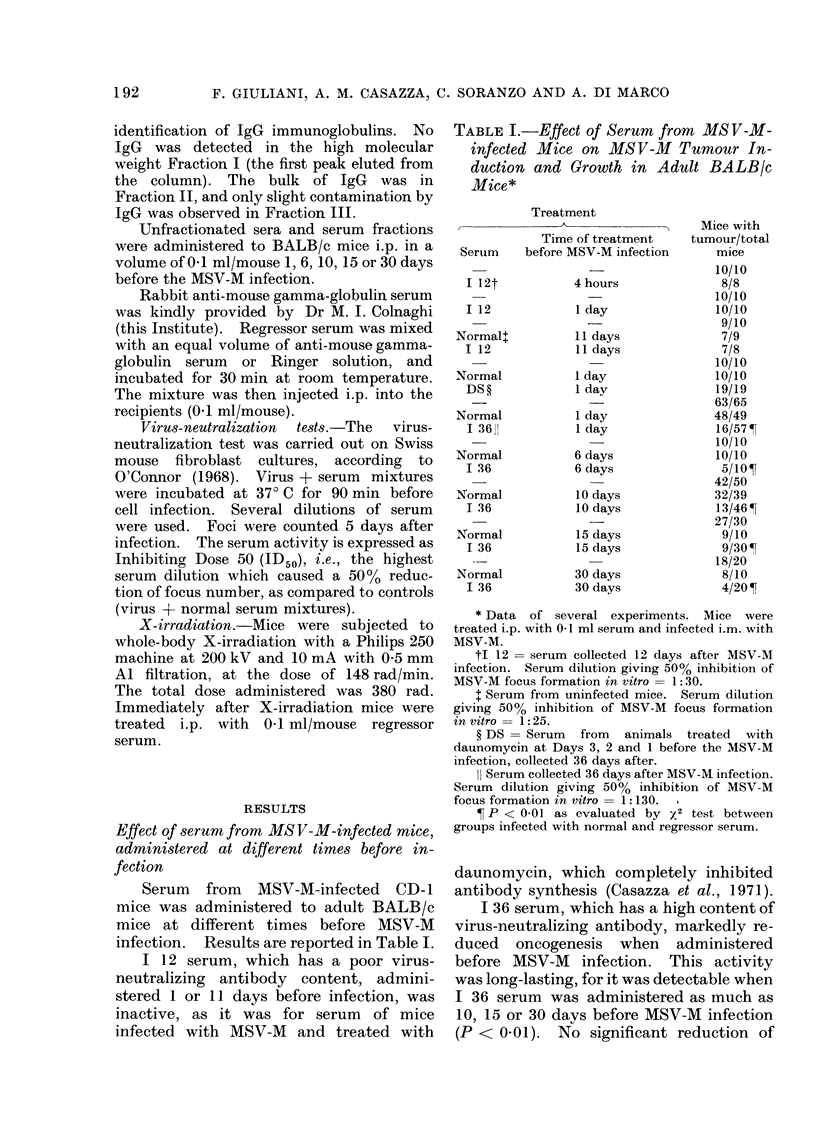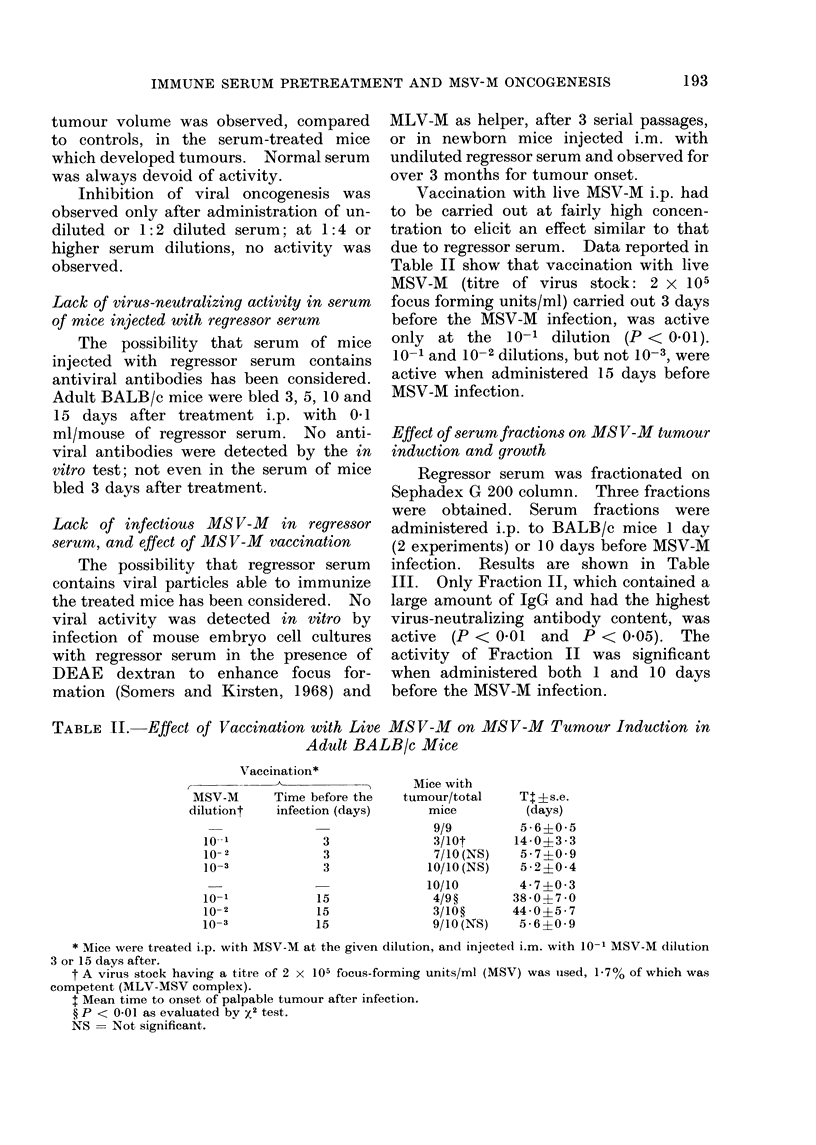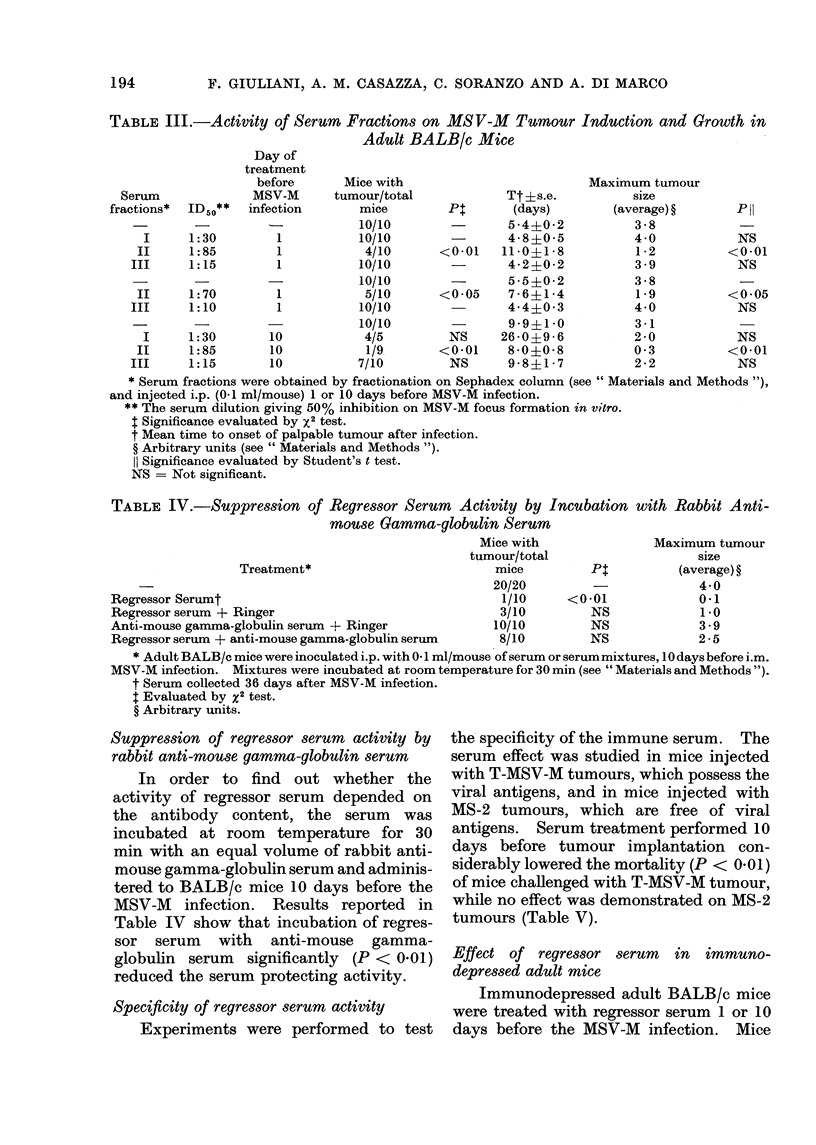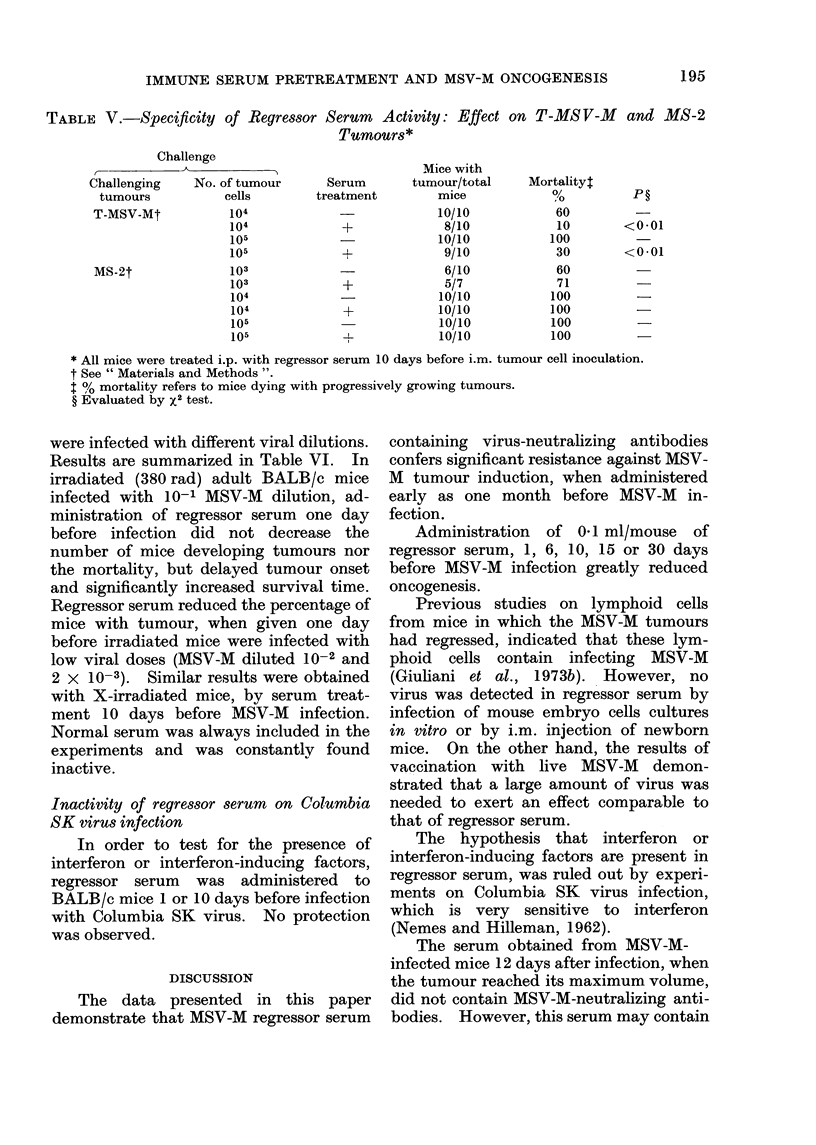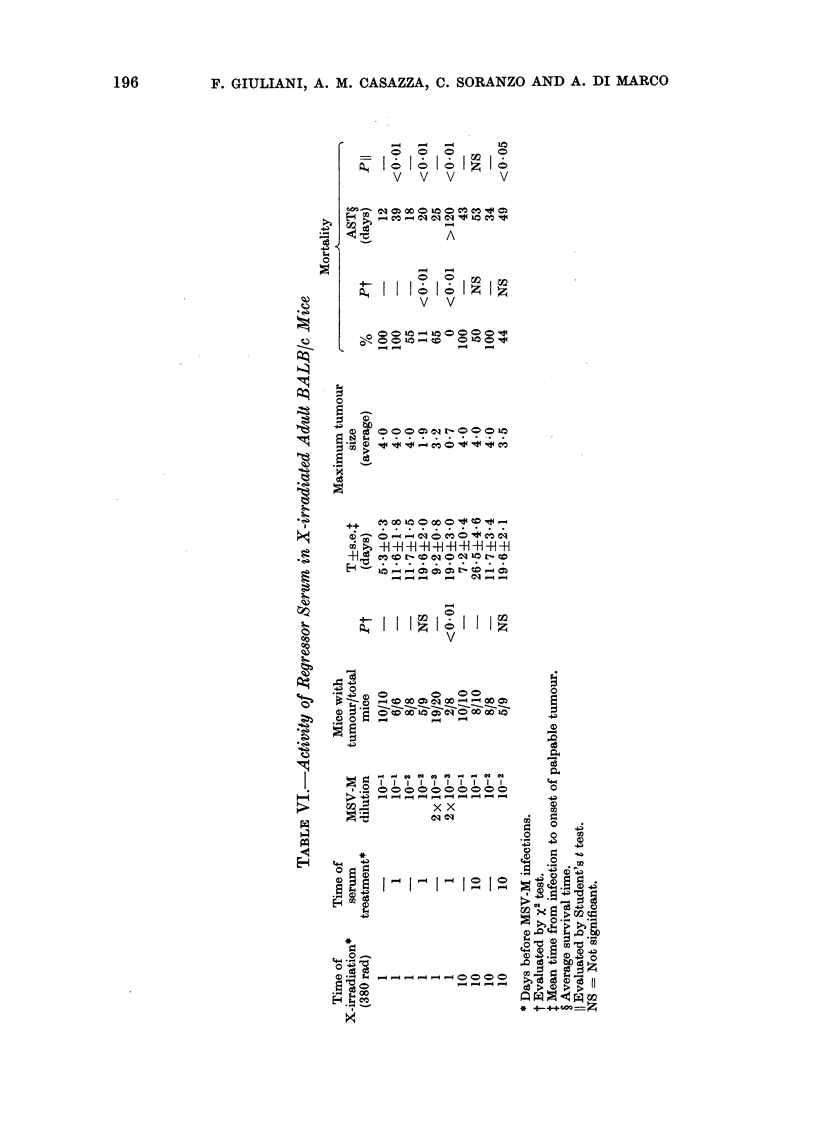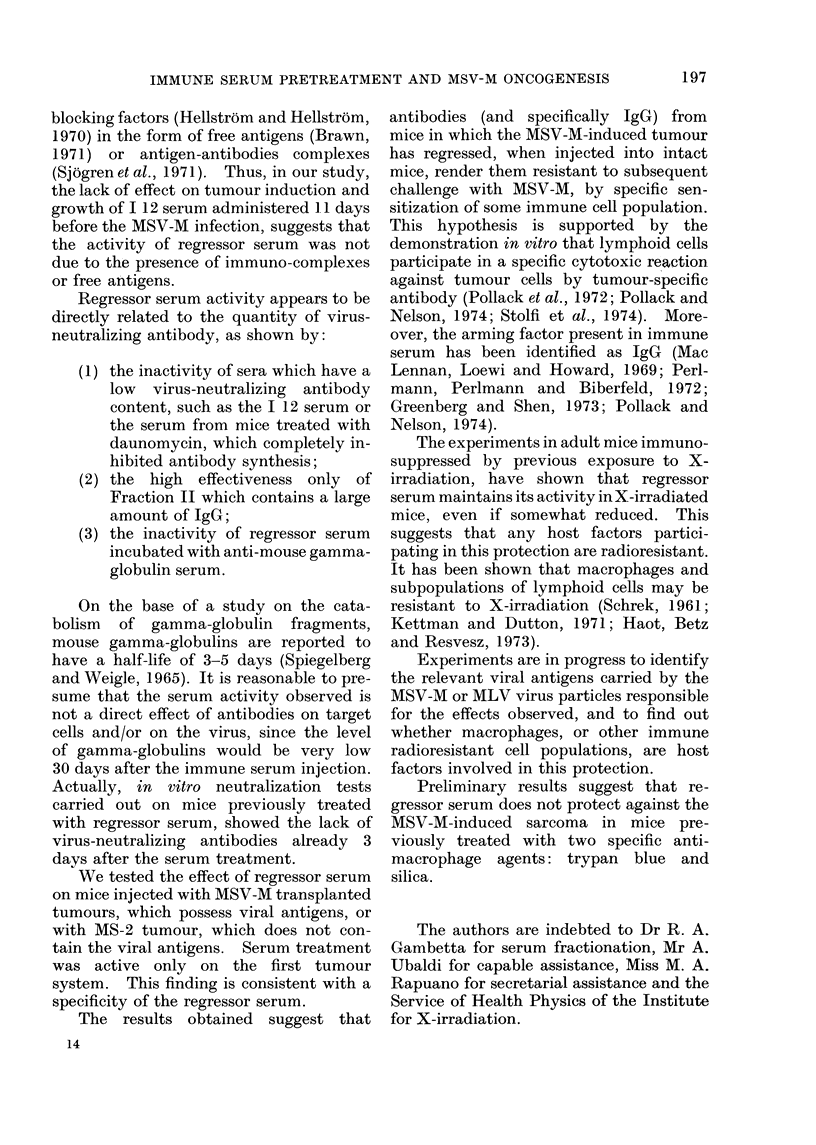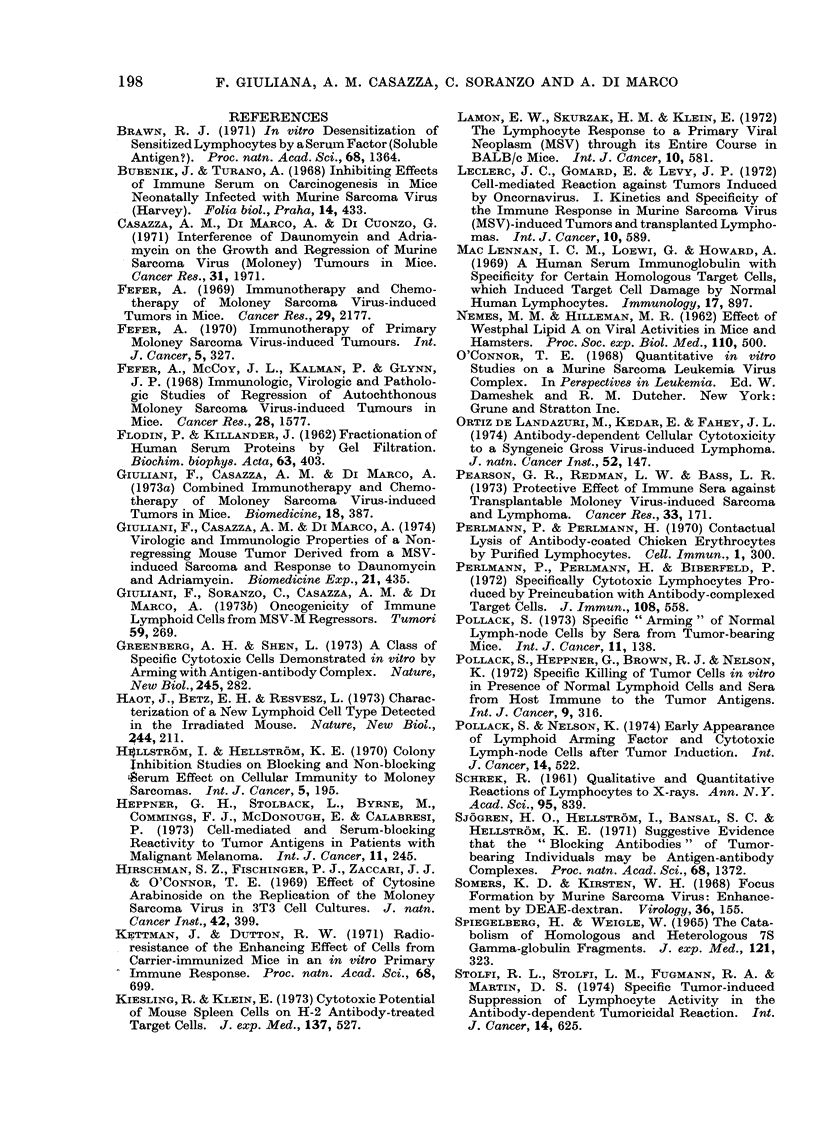# Effect of pretreatment with immune serum on murine sarcoma virus (Moloney) tumour induction and growth.

**DOI:** 10.1038/bjc.1977.26

**Published:** 1977-02

**Authors:** F. Guiliani, A. M. Casazza, C. Soranzo, A. Di Marco

## Abstract

Regressor serum from MSV-M-infected mice markedly reduced MSV-M oncogenesis when administered i.p. (0-1 ml/mouse) as much as 30 days before i.m. MSV-M infection in adult BALB/c mice. The regressor serum activity appeared to be directly dependent on the amount of IgG, as shown by: (1) inactivity of sera which have low virus-neutralizing antibody content; (2) high effectiveness only of the IgG serum fraction; (3) inactivity of regressor serum incubated with anti-mouse gamma-globulin serum. The regressor serum activity was specific and could not be ascribed to interferon or interferon-inducing factors, antigen-antibody complexes or free antigen. The activity was not suppressed by sublethal irradiation (380 rad) of recipient mice. These results suggest that the activity of regressor serum administered before MSV-M infection is mediated through sensitization of host cells which are not radiosensitive.


*Br. J. Cancer* (1977) **35**, 190

### EFFECT OF PRETREATMENT WITH IMMUNE SERUM ON MURINE SARCOMA VIRUS (MOLONEY) TUMOUR INDUCTION AND GROWTH*

F. GIULIANI, A. M. CASAZZA†, C. SORANZO AND A. DI MARCO

*From the Division of Experimental Oncology B, Istituto Nazionale per lo Studio e la Cura dei Tumori, Via G. Venezian* 1, 20133 *Milano*, Italy

Received 2 June 1976 Accepted 14 September 1976

**Summary.—Regressor serum from MSV-M-infected mice markedly reduced MSV-M oncogenesis when administered i.p. (0·1 ml/mouse) as much as 30 days before i.m. MSV-M infection in adult BALB/c mice. The regressor serum activity appeared to be directly dependent on the amount of IgG, as shown by: (1) inactivity of sera which have low virus-neutralizing antibody content; (2) high effectiveness only of the IgG serum fraction; (3) inactivity of regressor serum incubated with anti-mouse gamma-globulin serum. The regressor serum activity was specific and could not be ascribed to interferon or interferon-inducing factors, antigen-antibody complexes or free antigen. The activity was not suppressed by sublethal irradiation (380 rad) of recipient mice. These results suggest that the activity of regressor serum administered before MSV-M infection is mediated through sensitization of host cells which are not radiosensitive.**

THE TUMOUR induced in mice by Murine Sarcoma Virus (Moloney) (MSV-M) is a useful model for investigating the immunological mechanism involved in the regression or progression of virus-induced tumours. This tumour is known to regress spontaneously as a result of immunological host reactions (Fefer *et al.*, 1968). Mice in which the tumour has regressed have serum virus-neutralizing antibodies and lymphoid cells which are cytotoxic for Moloney sarcoma cells *in vitro* (Lamon, Skurzak and Klein, 1972; Leclerc, Gomard and Levy, 1972).

Inhibition of MSV-M-induced tumour growth by treatment with regressor serum or its 7S fraction, administered after infection, has been reported by several authors (Bubenik and Turano, 1968; Fefer, 1969; Fefer, 1970; Giuliani, Casazza and Di Marco, 1973*a*).

Moreover, regressor serum, or its 7S fraction, induced tumour regression in immunosuppressed mice bearing established MSV-M-induced tumours (Giuliani *et al.*, 1973*a*). Pearson, Redman and Bass (1973) have shown that regressor serum is active also when administered 3 days before challenge with tumour cells bearing the MSV-M antigens, and suggest that the antibody is not the only active factor in regressor serum, since the half-life of mouse immunoglobulin is 3–5 days (Spiegelberg and Weigle, 1965).

A synergistic effect between immune sera and normal lymphoid cells has been reported in a number of animal and human tumours (Perlmann and Perlmann, 1970; Heppner *et al.*, 1973; Kiesling and Klein, 1973; Ortiz de Landazuri, Kedar and Fahey, 1974). Sera from MSV-M tumour-bearing mice are specifically cytotoxic to tumour cells *in vitro*, when non-sensitized syngeneic lymphoid cells are added (Pollack *et al.*, 1972). Lymphoid cells from non-sensitized mice can also be

* Preliminary report presented to 11th International Cancer Congress, Florence, Italy, in October 1974.
† On leave from Farmitalia Research Institute, Milano, Italy.

sensitized *in vivo* by passively transferred serum from syngeneic MSV-M tumour-bearing donors (Pollack, 1973). The sensitized lymphoid cells are specifically cytotoxic to target tumour cells *in vitro*.

Data presented here show that the injection of serum from mice in which the MSV-M-induced tumour had completely regressed strongly inhibited MSV-M oncogenesis and tumour growth, even when the serum was injected as much as 30 days before the MSV-M infection. This activity is specific and directly related to the serum IgG content. We suggest that IgG may act by sensitizing host immune cells, shown not to be radiosensitive.

### MATERIALS AND METHODS

*Animals*.—Newborn or 4–6-week-old inbred BALB/c mice, and 16-day-old outbred Ha/ICR mice of the CD-1 line (received from Charles River, Calco, Italy) were used. For *in vitro* studies, secondary mouse embryo cell cultures from Swiss NIH mice bred in our laboratory were used.

*Virus*.—MSV-M, kindly provided by Dr J. B. Moloney (National Cancer Institute, Bethesda, Md., USA) was used for tumour induction in adult BALB/c or CD-1 mice as previously described (Casazza, Di Marco and Di Cuonzo, 1971). Mice were infected by inoculating intramuscularly (i.m.) 0·1 ml of $10^{-1}$ dilution of MSV-M preparation in the right hind leg (unless otherwise stated). The titre of the viral preparation, determined by the focus assay according to Hirschmann *et al.* (1969), was $2 \times 10^5$ focus-forming units/ml (MSV-M), 1·7% of which was competent (MLV-MSV complex). The percent of regression of MSV-induced tumours in outbred CD-1 mice was around 95%; in inbred BALB/c mice, the percent of tumour regression was around 60%. The BALB/c mice show also a significantly high incidence of tumour recurrence.

Columbia SK virus, an encephalomyocarditis virus, was kindly provided by Dr A. Fioretti (Farmitalia Research Laboratories, Nerviano, Italy). This virus is extremely sensitive to interferon (Nemes and Hilleman, 1962). All animals inoculated i.p. with $10^{-2}$ dilution ($10 \times LD_{50}$) of the Columbia SK virus preparation died within 10–11 days of infection.

*Tumours*.—The following tumours were used:

(a) primary tumours induced by MSV-M infection in adult BALB/c mice, as above described;
(b) a Moloney transplanted sarcoma (T-MSV-M) originally induced in BALB/c mice by i.m. infection with MSV-M. This tumour has been maintained and passed routinely in syngeneic hosts for over 2 years (Giuliani, Casazza and Di Marco, 1974);
(c) MS-2 tumours, obtained by i.m. injection in BALB/c mice of MS-2 cells (a cell line established *in vitro* in our laboratory by serial culturing of primary MSV-M tumours) and then maintained by *in vivo* passage in syngeneic host (Giuliani *et al.*, 1974).

The primary MSV-M and the T-MSV-M tumours possess the viral antigens and spontaneously regress, while MS-2 tumours do not exhibit the viral antigens, grow progressively and kill the host (Giuliani *et al.*, 1974).

*Tumour measurements*.—Mice were observed daily, and tumour growth was determined and graded on an arbitrary scale of + 1 through + 4 as previously described (Casazza *et al.*, 1971).

*Sera*.—CD-1 mice were used as serum donors, as they were available in large numbers. Pools of sera were obtained by cardiac puncture from uninfected (normal) or MSV-M-infected mice, 12 (I 12) or 36 (I 36) days after infection, or from mice treated with daunomycin beforehand, as previously described (Giuliani *et al.*, 1973*a*).

Twelve days after infection the tumour reached the maximal volume, and after 36 days it had completely regressed. The titre of virus-neutralizing antibodies was maximal in I 36 serum, but very low in I 12 serum as previously observed (Casazza *et al.*, 1971). Sera were heat-inactivated at 56° C for 30 min and stored at −20°C.

Serum fractions were obtained by separation on Sephadex G 200 column, according to Flodin and Killander (1962). Protein elution was detected by continuous monitoring of the effluent at 280 nm. Three fractions were obtained. Each serum fraction was concentrated by ultrafiltration up to the volume of the original serum sample. Agar-gel immunoelectrophoretic analysis was used for identification of IgG immunoglobulins. No IgG was detected in the high molecular weight Fraction I (the first peak eluted from the column). The bulk of IgG was in Fraction II, and only slight contamination by IgG was observed in Fraction III.

Unfractionated sera and serum fractions were administered to BALB/c mice i.p. in a volume of 0·1 ml/mouse 1, 6, 10, 15 or 30 days before the MSV-M infection.

Rabbit anti-mouse gamma-globulin serum was kindly provided by Dr M. I. Colnaghi (this Institute). Regressor serum was mixed with an equal volume of anti-mouse gamma-globulin serum or Ringer solution, and incubated for 30 min at room temperature. The mixture was then injected i.p. into the recipients (0·1 ml/mouse).

*Virus-neutralization tests.*—The virus-neutralization test was carried out on Swiss mouse fibroblast cultures, according to O'Connor (1968). Virus + serum mixtures were incubated at 37° C for 90 min before cell infection. Several dilutions of serum were used. Foci were counted 5 days after infection. The serum activity is expressed as Inhibiting Dose 50 ($ID_{50}$), *i.e.*, the highest serum dilution which caused a 50% reduction of focus number, as compared to controls (virus + normal serum mixtures).

*X-irradiation.*—Mice were subjected to whole-body X-irradiation with a Philips 250 machine at 200 kV and 10 mA with 0·5 mm Al filtration, at the dose of 148 rad/min. The total dose administered was 380 rad. Immediately after X-irradiation mice were treated i.p. with 0·1 ml/mouse regressor serum.

TABLE I.—*Effect of Serum from MSV-M-infected Mice on MSV-M Tumour Induction and Growth in Adult BALB/c Mice**

| Treatment: Serum | Treatment: Time of treatment before MSV-M infection | Mice with tumour/total mice |
|---|---|---|
| — | — | 10/10 |
| I 12† | 4 hours | 8/8 |
| — | — | 10/10 |
| I 12 | 1 day | 10/10 |
| — | — | 9/10 |
| Normal‡ | 11 days | 7/9 |
| I 12 | 11 days | 7/8 |
| — | — | 10/10 |
| Normal | 1 day | 10/10 |
| DS§ | 1 day | 19/19 |
| — | — | 63/65 |
| Normal | 1 day | 48/49 |
| I 36‖ | 1 day | 16/57¶ |
| — | — | 10/10 |
| Normal | 6 days | 10/10 |
| I 36 | 6 days | 5/10¶ |
| — | — | 42/50 |
| Normal | 10 days | 32/39 |
| I 36 | 10 days | 13/46¶ |
| — | — | 27/30 |
| Normal | 15 days | 9/10 |
| I 36 | 15 days | 9/30¶ |
| — | — | 18/20 |
| Normal | 30 days | 8/10 |
| I 36 | 30 days | 4/20¶ |

* Data of several experiments. Mice were treated i.p. with 0·1 ml serum and infected i.m. with MSV-M.

†I 12 = serum collected 12 days after MSV-M infection. Serum dilution giving 50% inhibition of MSV-M focus formation *in vitro* = 1:30.

‡ Serum from uninfected mice. Serum dilution giving 50% inhibition of MSV-M focus formation *in vitro* = 1:25.

§ DS = Serum from animals treated with daunomycin at Days 3, 2 and 1 before the MSV-M infection, collected 36 days after.

‖ Serum collected 36 days after MSV-M infection. Serum dilution giving 50% inhibition of MSV-M focus formation *in vitro* = 1:130.

¶ $P < 0{\cdot}01$ as evaluated by $\chi^2$ test between groups infected with normal and regressor serum.

### RESULTS

### *Effect of serum from MSV-M-infected mice, administered at different times before infection*

Serum from MSV-M-infected CD-1 mice was administered to adult BALB/c mice at different times before MSV-M infection. Results are reported in Table I.

I 12 serum, which has a poor virus-neutralizing antibody content, administered 1 or 11 days before infection, was inactive, as it was for serum of mice infected with MSV-M and treated with daunomycin, which completely inhibited antibody synthesis (Casazza *et al.*, 1971).

I 36 serum, which has a high content of virus-neutralizing antibody, markedly reduced oncogenesis when administered before MSV-M infection. This activity was long-lasting, for it was detectable when I 36 serum was administered as much as 10, 15 or 30 days before MSV-M infection ($P < 0{\cdot}01$). No significant reduction of tumour volume was observed, compared to controls, in the serum-treated mice which developed tumours. Normal serum was always devoid of activity.

Inhibition of viral oncogenesis was observed only after administration of undiluted or 1:2 diluted serum; at 1:4 or higher serum dilutions, no activity was observed.

*Lack of virus-neutralizing activity in serum of mice injected with regressor serum*

The possibility that serum of mice injected with regressor serum contains antiviral antibodies has been considered. Adult BALB/c mice were bled 3, 5, 10 and 15 days after treatment i.p. with 0·1 ml/mouse of regressor serum. No antiviral antibodies were detected by the *in vitro* test; not even in the serum of mice bled 3 days after treatment.

*Lack of infectious MSV-M in regressor serum, and effect of MSV-M vaccination*

The possibility that regressor serum contains viral particles able to immunize the treated mice has been considered. No viral activity was detected *in vitro* by infection of mouse embryo cell cultures with regressor serum in the presence of DEAE dextran to enhance focus formation (Somers and Kirsten, 1968) and MLV-M as helper, after 3 serial passages, or in newborn mice injected i.m. with undiluted regressor serum and observed for over 3 months for tumour onset.

Vaccination with live MSV-M i.p. had to be carried out at fairly high concentration to elicit an effect similar to that due to regressor serum. Data reported in Table II show that vaccination with live MSV-M (titre of virus stock: $2 \times 10^5$ focus forming units/ml) carried out 3 days before the MSV-M infection, was active only at the $10^{-1}$ dilution ($P < 0{\cdot}01$). $10^{-1}$ and $10^{-2}$ dilutions, but not $10^{-3}$, were active when administered 15 days before MSV-M infection.

*Effect of serum fractions on MSV-M tumour induction and growth*

Regressor serum was fractionated on Sephadex G 200 column. Three fractions were obtained. Serum fractions were administered i.p. to BALB/c mice 1 day (2 experiments) or 10 days before MSV-M infection. Results are shown in Table III. Only Fraction II, which contained a large amount of IgG and had the highest virus-neutralizing antibody content, was active ($P < 0{\cdot}01$ and $P < 0{\cdot}05$). The activity of Fraction II was significant when administered both 1 and 10 days before the MSV-M infection.

TABLE II.—*Effect of Vaccination with Live MSV-M on MSV-M Tumour Induction in Adult BALB/c Mice*

| Vaccination* | | | |
|---|---|---|---|
| MSV-M dilution† | Time before the infection (days) | Mice with tumour/total mice | T‡ ± s.e. (days) |
| — | — | 9/9 | 5·6 ± 0·5 |
| $10^{-1}$ | 3 | 3/10† | 14·0 ± 3·3 |
| $10^{-2}$ | 3 | 7/10 (NS) | 5·7 ± 0·9 |
| $10^{-3}$ | 3 | 10/10 (NS) | 5·2 ± 0·4 |
| — | — | 10/10 | 4·7 ± 0·3 |
| $10^{-1}$ | 15 | 4/9§ | 38·0 ± 7·0 |
| $10^{-2}$ | 15 | 3/10§ | 44·0 ± 5·7 |
| $10^{-3}$ | 15 | 9/10 (NS) | 5·6 ± 0·9 |

* Mice were treated i.p. with MSV-M at the given dilution, and injected i.m. with $10^{-1}$ MSV-M dilution 3 or 15 days after.

† A virus stock having a titre of $2 \times 10^5$ focus-forming units/ml (MSV) was used, 1·7% of which was competent (MLV-MSV complex).

‡ Mean time to onset of palpable tumour after infection.

§ $P < 0{\cdot}01$ as evaluated by $\chi^2$ test.

NS = Not significant.

TABLE III.—*Activity of Serum Fractions on MSV-M Tumour Induction and Growth in Adult BALB/c Mice*

| Serum fractions* | $ID_{50}$** | Day of treatment before MSV-M infection | Mice with tumour/total mice | P‡ | T†±s.e. (days) | Maximum tumour size (average)§ | P‖ |
|---|---|---|---|---|---|---|---|
| — | — | — | 10/10 | — | 5·4±0·2 | 3·8 | — |
| I | 1:30 | 1 | 10/10 | — | 4·8±0·5 | 4·0 | NS |
| II | 1:85 | 1 | 4/10 | <0·01 | 11·0±1·8 | 1·2 | <0·01 |
| III | 1:15 | 1 | 10/10 | — | 4·2±0·2 | 3·9 | NS |
| — | — | — | 10/10 | — | 5·5±0·2 | 3·8 | — |
| II | 1:70 | 1 | 5/10 | <0·05 | 7·6±1·4 | 1·9 | <0·05 |
| III | 1:10 | 1 | 10/10 | — | 4·4±0·3 | 4·0 | NS |
| — | — | — | 10/10 | — | 9·9±1·0 | 3·1 | — |
| I | 1:30 | 10 | 4/5 | NS | 26·0±9·6 | 2·0 | NS |
| II | 1:85 | 10 | 1/9 | <0·01 | 8·0±0·8 | 0·3 | <0·01 |
| III | 1:15 | 10 | 7/10 | NS | 9·8±1·7 | 2·2 | NS |

* Serum fractions were obtained by fractionation on Sephadex column (see " Materials and Methods "), and injected i.p. (0·1 ml/mouse) 1 or 10 days before MSV-M infection.
** The serum dilution giving 50% inhibition on MSV-M focus formation *in vitro*.
‡ Significance evaluated by $\chi^2$ test.
† Mean time to onset of palpable tumour after infection.
§ Arbitrary units (see " Materials and Methods ").
‖ Significance evaluated by Student's *t* test.
NS = Not significant.

TABLE IV.—*Suppression of Regressor Serum Activity by Incubation with Rabbit Anti-mouse Gamma-globulin Serum*

| Treatment* | Mice with tumour/total mice | P‡ | Maximum tumour size (average)§ |
|---|---|---|---|
| — | 20/20 | — | 4·0 |
| Regressor Serum† | 1/10 | <0·01 | 0·1 |
| Regressor serum + Ringer | 3/10 | NS | 1·0 |
| Anti-mouse gamma-globulin serum + Ringer | 10/10 | NS | 3·9 |
| Regressor serum + anti-mouse gamma-globulin serum | 8/10 | NS | 2·5 |

* Adult BALB/c mice were inoculated i.p. with 0·1 ml/mouse of serum or serum mixtures, 10 days before i.m. MSV-M infection. Mixtures were incubated at room temperature for 30 min (see " Materials and Methods ").
† Serum collected 36 days after MSV-M infection.
‡ Evaluated by $\chi^2$ test.
§ Arbitrary units.

*Suppression of regressor serum activity by rabbit anti-mouse gamma-globulin serum*

In order to find out whether the activity of regressor serum depended on the antibody content, the serum was incubated at room temperature for 30 min with an equal volume of rabbit anti-mouse gamma-globulin serum and administered to BALB/c mice 10 days before the MSV-M infection. Results reported in Table IV show that incubation of regressor serum with anti-mouse gamma-globulin serum significantly ($P < 0{\cdot}01$) reduced the serum protecting activity.

*Specificity of regressor serum activity*

Experiments were performed to test the specificity of the immune serum. The serum effect was studied in mice injected with T-MSV-M tumours, which possess the viral antigens, and in mice injected with MS-2 tumours, which are free of viral antigens. Serum treatment performed 10 days before tumour implantation considerably lowered the mortality ($P < 0{\cdot}01$) of mice challenged with T-MSV-M tumour, while no effect was demonstrated on MS-2 tumours (Table V).

*Effect of regressor serum in immunodepressed adult mice*

Immunodepressed adult BALB/c mice were treated with regressor serum 1 or 10 days before the MSV-M infection. Mice

TABLE V.—*Specificity of Regressor Serum Activity: Effect on T-MSV-M and MS-2 Tumours**

| Challenge | | | | | |
|---|---|---|---|---|---|
| Challenging tumours | No. of tumour cells | Serum treatment | Mice with tumour/total mice | Mortality‡ % | P§ |
| T-MSV-M† | $10^4$ | — | 10/10 | 60 | — |
| | $10^4$ | + | 8/10 | 10 | <0·01 |
| | $10^5$ | — | 10/10 | 100 | — |
| | $10^5$ | + | 9/10 | 30 | <0·01 |
| MS-2† | $10^3$ | — | 6/10 | 60 | — |
| | $10^3$ | + | 5/7 | 71 | — |
| | $10^4$ | — | 10/10 | 100 | — |
| | $10^4$ | + | 10/10 | 100 | — |
| | $10^5$ | — | 10/10 | 100 | — |
| | $10^5$ | + | 10/10 | 100 | — |

* All mice were treated i.p. with regressor serum 10 days before i.m. tumour cell inoculation.
† See " Materials and Methods ".
‡ % mortality refers to mice dying with progressively growing tumours.
§ Evaluated by $\chi^2$ test.

were infected with different viral dilutions. Results are summarized in Table VI. In irradiated (380 rad) adult BALB/c mice infected with $10^{-1}$ MSV-M dilution, administration of regressor serum one day before infection did not decrease the number of mice developing tumours nor the mortality, but delayed tumour onset and significantly increased survival time. Regressor serum reduced the percentage of mice with tumour, when given one day before irradiated mice were infected with low viral doses (MSV-M diluted $10^{-2}$ and $2 \times 10^{-3}$). Similar results were obtained with X-irradiated mice, by serum treatment 10 days before MSV-M infection. Normal serum was always included in the experiments and was constantly found inactive.

*Inactivity of regressor serum on Columbia SK virus infection*

In order to test for the presence of interferon or interferon-inducing factors, regressor serum was administered to BALB/c mice 1 or 10 days before infection with Columbia SK virus. No protection was observed.

### DISCUSSION

The data presented in this paper demonstrate that MSV-M regressor serum containing virus-neutralizing antibodies confers significant resistance against MSV-M tumour induction, when administered early as one month before MSV-M infection.

Administration of 0·1 ml/mouse of regressor serum, 1, 6, 10, 15 or 30 days before MSV-M infection greatly reduced oncogenesis.

Previous studies on lymphoid cells from mice in which the MSV-M tumours had regressed, indicated that these lymphoid cells contain infecting MSV-M (Giuliani *et al.*, 1973*b*). However, no virus was detected in regressor serum by infection of mouse embryo cells cultures *in vitro* or by i.m. injection of newborn mice. On the other hand, the results of vaccination with live MSV-M demonstrated that a large amount of virus was needed to exert an effect comparable to that of regressor serum.

The hypothesis that interferon or interferon-inducing factors are present in regressor serum, was ruled out by experiments on Columbia SK virus infection, which is very sensitive to interferon (Nemes and Hilleman, 1962).

The serum obtained from MSV-M-infected mice 12 days after infection, when the tumour reached its maximum volume, did not contain MSV-M-neutralizing antibodies. However, this serum may contain

TABLE VI.—*Activity of Regressor Serum in X-irradiated Adult BALB/c Mice*

| Time of X-irradiation* (380 rad) | Time of serum treatment* | MSV-M dilution | Mice with tumour/total mice | P† | T ± s.e.‡ (days) | Maximum tumour size (average) | Mortality % | Mortality P† | Mortality AST§ (days) | Mortality P‖ |
|---|---|---|---|---|---|---|---|---|---|---|
| 1 | — | $10^{-1}$ | 10/10 | — | 5·3±0·3 | 4·0 | 100 | — | 12 | — |
| 1 | 1 | $10^{-1}$ | 6/6 | — | 11·6±1·8 | 4·0 | 100 | — | 39 | <0·01 |
| 1 | — | $10^{-2}$ | 8/8 | — | 11·7±1·5 | 4·0 | 55 | — | 18 | — |
| 1 | 1 | $10^{-2}$ | 5/9 | NS | 19·6±2·0 | 1·9 | 11 | <0·01 | 20 | <0·01 |
| 1 | — | $2\times10^{-3}$ | 19/20 | — | 9·2±0·8 | 3·2 | 65 | — | 25 | — |
| 1 | 1 | $2\times10^{-3}$ | 2/8 | <0·01 | 19·0±3·0 | 0·7 | 0 | <0·01 | >120 | <0·01 |
| 10 | — | $10^{-1}$ | 10/10 | — | 7·2±0·4 | 4·0 | 100 | — | 43 | — |
| 10 | 10 | $10^{-1}$ | 8/10 | — | 26·5±4·6 | 4·0 | 50 | NS | 53 | NS |
| 10 | — | $10^{-2}$ | 8/8 | — | 11·7±3·4 | 4·0 | 100 | — | 34 | — |
| 10 | 10 | $10^{-2}$ | 5/9 | NS | 19·6±2·1 | 3·5 | 44 | NS | 49 | <0·05 |

* Days before MSV-M infections.

† Evaluated by $\chi^2$ test.

‡ Mean time from infection to onset of palpable tumour.

§ Average survival time.

‖ Evaluated by Student's *t* test.

NS = Not significant.

blocking factors (Hellström and Hellström, 1970) in the form of free antigens (Brawn, 1971) or antigen-antibodies complexes (Sjögren *et al.*, 1971). Thus, in our study, the lack of effect on tumour induction and growth of I 12 serum administered 11 days before the MSV-M infection, suggests that the activity of regressor serum was not due to the presence of immuno-complexes or free antigens.

Regressor serum activity appears to be directly related to the quantity of virus-neutralizing antibody, as shown by:

(1) the inactivity of sera which have a low virus-neutralizing antibody content, such as the I 12 serum or the serum from mice treated with daunomycin, which completely inhibited antibody synthesis;
(2) the high effectiveness only of Fraction II which contains a large amount of IgG;
(3) the inactivity of regressor serum incubated with anti-mouse gamma-globulin serum.

On the base of a study on the catabolism of gamma-globulin fragments, mouse gamma-globulins are reported to have a half-life of 3–5 days (Spiegelberg and Weigle, 1965). It is reasonable to presume that the serum activity observed is not a direct effect of antibodies on target cells and/or on the virus, since the level of gamma-globulins would be very low 30 days after the immune serum injection. Actually, *in vitro* neutralization tests carried out on mice previously treated with regressor serum, showed the lack of virus-neutralizing antibodies already 3 days after the serum treatment.

We tested the effect of regressor serum on mice injected with MSV-M transplanted tumours, which possess viral antigens, or with MS-2 tumour, which does not contain the viral antigens. Serum treatment was active only on the first tumour system. This finding is consistent with a specificity of the regressor serum.

The results obtained suggest that antibodies (and specifically IgG) from mice in which the MSV-M-induced tumour has regressed, when injected into intact mice, render them resistant to subsequent challenge with MSV-M, by specific sensitization of some immune cell population. This hypothesis is supported by the demonstration *in vitro* that lymphoid cells participate in a specific cytotoxic reaction against tumour cells by tumour-specific antibody (Pollack *et al.*, 1972; Pollack and Nelson, 1974; Stolfi et *al.*, 1974). Moreover, the arming factor present in immune serum has been identified as IgG (Mac Lennan, Loewi and Howard, 1969; Perlmann, Perlmann and Biberfeld, 1972; Greenberg and Shen, 1973; Pollack and Nelson, 1974).

The experiments in adult mice immunosuppressed by previous exposure to X-irradiation, have shown that regressor serum maintains its activity in X-irradiated mice, even if somewhat reduced. This suggests that any host factors participating in this protection are radioresistant. It has been shown that macrophages and subpopulations of lymphoid cells may be resistant to X-irradiation (Schrek, 1961; Kettman and Dutton, 1971; Haot, Betz and Resvesz, 1973).

Experiments are in progress to identify the relevant viral antigens carried by the MSV-M or MLV virus particles responsible for the effects observed, and to find out whether macrophages, or other immune radioresistant cell populations, are host factors involved in this protection.

Preliminary results suggest that regressor serum does not protect against the MSV-M-induced sarcoma in mice previously treated with two specific anti-macrophage agents: trypan blue and silica.

The authors are indebted to Dr R. A. Gambetta for serum fractionation, Mr A. Ubaldi for capable assistance, Miss M. A. Rapuano for secretarial assistance and the Service of Health Physics of the Institute for X-irradiation.

### REFERENCES

BRAWN, R. J. (1971) *In vitro* Desensitization of Sensitized Lymphocytes by a Serum Factor (Soluble Antigen?). *Proc. natn. Acad. Sci.*, **68**, 1364.

BUBENIK, J. & TURANO, A. (1968) Inhibiting Effects of Immune Serum on Carcinogenesis in Mice Neonatally Infected with Murine Sarcoma Virus (Harvey). *Folia biol., Praha*, **14**, 433.

CASAZZA, A. M., DI MARCO, A. & DI CUONZO, G. (1971) Interference of Daunomycin and Adriamycin on the Growth and Regression of Murine Sarcoma Virus (Moloney) Tumours in Mice. *Cancer Res.*, **31**, 1971.

FEFER, A. (1969) Immunotherapy and Chemotherapy of Moloney Sarcoma Virus-induced Tumors in Mice. *Cancer Res.*, **29**, 2177.

FEFER, A. (1970) Immunotherapy of Primary Moloney Sarcoma Virus-induced Tumours. *Int. J. Cancer*, **5**, 327.

FEFER, A., MCCOY, J. L., KALMAN, P. & GLYNN, J. P. (1968) Immunologic, Virologic and Pathologic Studies of Regression of Autochthonous Moloney Sarcoma Virus-induced Tumours in Mice. *Cancer Res.*, **28**, 1577.

FLODIN, P. & KILLANDER, J. (1962) Fractionation of Human Serum Proteins by Gel Filtration. *Biochim. biophys. Acta*, **63**, 403.

GIULIANI, F., CASAZZA, A. M. & DI MARCO, A. (1973*a*) Combined Immunotherapy and Chemotherapy of Moloney Sarcoma Virus-induced Tumors in Mice. *Biomedicine*, **18**, 387.

GIULIANI, F., CASAZZA, A. M. & DI MARCO, A. (1974) Virologic and Immunologic Properties of a Non-regressing Mouse Tumor Derived from a MSV-induced Sarcoma and Response to Daunomycin and Adriamycin. *Biomedicine Exp.*, **21**, 435.

GIULIANI, F., SORANZO, C., CASAZZA, A. M. & DI MARCO, A. (1973*b*) Oncogenicity of Immune Lymphoid Cells from MSV-M Regressors. *Tumori* **59**, 269.

GREENBERG, A. H. & SHEN, L. (1973) A Class of Specific Cytotoxic Cells Demonstrated *in vitro* by Arming with Antigen-antibody Complex. *Nature, New Biol.*, **245**, 282.

HAOT, J., BETZ, E. H. & RESVESZ, L. (1973) Characterization of a New Lymphoid Cell Type Detected in the Irradiated Mouse. *Nature, New Biol.*, **244**, 211.

HELLSTRÖM, I. & HELLSTRÖM, K. E. (1970) Colony Inhibition Studies on Blocking and Non-blocking Serum Effect on Cellular Immunity to Moloney Sarcomas. *Int. J. Cancer*, **5**, 195.

HEPPNER, G. H., STOLBACK, L., BYRNE, M., COMMINGS, F. J., MCDONOUGH, E. & CALABRESI, P. (1973) Cell-mediated and Serum-blocking Reactivity to Tumor Antigens in Patients with Malignant Melanoma. *Int. J. Cancer*, **11**, 245.

HIRSCHMAN, S. Z., FISCHINGER, P. J., ZACCARI, J. J. & O'CONNOR, T. E. (1969) Effect of Cytosine Arabinoside on the Replication of the Moloney Sarcoma Virus in 3T3 Cell Cultures. *J. natn. Cancer Inst.*, **42**, 399.

KETTMAN, J. & DUTTON, R. W. (1971) Radioresistance of the Enhancing Effect of Cells from Carrier-immunized Mice in an *in vitro* Primary Immune Response. *Proc. natn. Acad. Sci.*, **68**, 699.

KIESLING, R. & KLEIN, E. (1973) Cytotoxic Potential of Mouse Spleen Cells on H-2 Antibody-treated Target Cells. *J. exp. Med.*, **137**, 527.

LAMON, E. W., SKURZAK, H. M. & KLEIN, E. (1972) The Lymphocyte Response to a Primary Viral Neoplasm (MSV) through its Entire Course in BALB/c Mice. *Int. J. Cancer*, **10**, 581.

LECLERC, J. C., GOMARD, E. & LEVY, J. P. (1972) Cell-mediated Reaction against Tumors Induced by Oncornavirus. I. Kinetics and Specificity of the Immune Response in Murine Sarcoma Virus (MSV)-induced Tumors and transplanted Lymphomas. *Int. J. Cancer*, **10**, 589.

MAC LENNAN, I. C. M., LOEWI, G. & HOWARD, A. (1969) A Human Serum Immunoglobulin with Specificity for Certain Homologous Target Cells, which Induced Target Cell Damage by Normal Human Lymphocytes. *Immunology*, **17**, 897.

NEMES, M. M. & HILLEMAN, M. R. (1962) Effect of Westphal Lipid A on Viral Activities in Mice and Hamsters. *Proc. Soc. exp. Biol. Med.*, **110**, 500.

O'CONNOR, T. E. (1968) Quantitative *in vitro* Studies on a Murine Sarcoma Leukemia Virus Complex. In *Perspectives in Leukemia*. Ed. W. Dameshek and R. M. Dutcher. New York: Grune and Stratton Inc.

ORTIZ DE LANDAZURI, M., KEDAR, E. & FAHEY, J. L. (1974) Antibody-dependent Cellular Cytotoxicity to a Syngeneic Gross Virus-induced Lymphoma. *J. natn. Cancer Inst.*, **52**, 147.

PEARSON, G. R., REDMAN, L. W. & BASS, L. R. (1973) Protective Effect of Immune Sera against Transplantable Moloney Virus-induced Sarcoma and Lymphoma. *Cancer Res.*, **33**, 171.

PERLMANN, P. & PERLMANN, H. (1970) Contactual Lysis of Antibody-coated Chicken Erythrocytes by Purified Lymphocytes. *Cell. Immun.*, **1**, 300.

PERLMANN, P., PERLMANN, H. & BIBERFELD, P. (1972) Specifically Cytotoxic Lymphocytes Produced by Preincubation with Antibody-complexed Target Cells. *J. Immun.*, **108**, 558.

POLLACK, S. (1973) Specific "Arming" of Normal Lymph-node Cells by Sera from Tumor-bearing Mice. *Int. J. Cancer*, **11**, 138.

POLLACK, S., HEPPNER, G., BROWN, R. J. & NELSON, K. (1972) Specific Killing of Tumor Cells *in vitro* in Presence of Normal Lymphoid Cells and Sera from Host Immune to the Tumor Antigens. *Int. J. Cancer*, **9**, 316.

POLLACK, S. & NELSON, K. (1974) Early Appearance of Lymphoid Arming Factor and Cytotoxic Lymph-node Cells after Tumor Induction. *Int. J. Cancer*, **14**, 522.

SCHREK, R. (1961) Qualitative and Quantitative Reactions of Lymphocytes to X-rays. *Ann. N.Y. Acad. Sci.*, **95**, 839.

SJÖGREN, H. O., HELLSTRÖM, I., BANSAL, S. C. & HELLSTRÖM, K. E. (1971) Suggestive Evidence that the "Blocking Antibodies" of Tumor-bearing Individuals may be Antigen-antibody Complexes. *Proc. natn. Acad. Sci.*, **68**, 1372.

SOMERS, K. D. & KIRSTEN, W. H. (1968) Focus Formation by Murine Sarcoma Virus: Enhancement by DEAE-dextran. *Virology*, **36**, 155.

SPIEGELBERG, H. & WEIGLE, W. (1965) The Catabolism of Homologous and Heterologous 7S Gamma-globulin Fragments. *J. exp. Med.*, **121**, 323.

STOLFI, R. L., STOLFI, L. M., FUGMANN, R. A. & MARTIN, D. S. (1974) Specific Tumor-induced Suppression of Lymphocyte Activity in the Antibody-dependent Tumoricidal Reaction. *Int. J. Cancer*, **14**, 625.